# DigiBete, a Novel Chatbot to Support Transition to Adult Care of Young People/Young Adults With Type 1 Diabetes Mellitus: Outcomes From a Prospective, Multimethod, Nonrandomized Feasibility and Acceptability Study

**DOI:** 10.2196/74032

**Published:** 2025-07-23

**Authors:** Veronica Swallow, Janet Horsman, Eliza Mazlan, Fiona Campbell, Reza Zaidi, Madeleine Julian, Jacob Branchflower, Jackie Martin-Kerry, Helen Monks, Astha Soni, Alison Rodriguez, Rob Julian, Paul Dimitri

**Affiliations:** 1 Centre for Applied Health & Social Care Research, Sheffield Hallam University, Howard St, Sheffield City Centre, Sheffield, Sheffield, S1 1WB, United Kingdom, +44 (0)114 225 5555; 2 Children's Services, Leeds Teaching Hospitals NHS Trust, Leeds, United Kingdom; 3 Royal Liverpool University Hospital, Liverpool Womens NHS Foundation Trust, Liverpool, United Kingdom; 4 DigiBete, Leeds, United Kingdom; 5 National Institute for Health and Care Research HealthTech Research Centre, Sheffield Children's NHS Foundation Trust, Sheffield, United Kingdom; 6 College of Life Sciences, University of Leicester, Leicester, United Kingdom; 7 School of Healthcare, University of Leeds, Leeds, United Kingdom

**Keywords:** digital health, chatbot, homecare, young people and young adults, feasibility study, multimethods, young people, young adults

## Abstract

**Background:**

Transition to adult health care for young people and young adults (YP/YA) with type 1 diabetes mellitus (T1DM) starts around 11 years of age, but transition services may not meet their needs. A combination of self-management support digital health technologies exists, but no supportive chatbots with components to help YP/YA with T1DM were identified.

**Objective:**

The aims of this study were to (1) evaluate the novel DigiBete Chatbot, the first user-led, developmentally appropriate, clinically approved transition chatbot for YP/YA with T1DM from four English diabetes services and (2) assess the feasibility of a future trial of the chatbot.

**Methods:**

In a prospective, multimethod, nonrandomized feasibility and acceptability study in the UK National Health Service, YP/YA with T1DM from 4 hospital diabetes clinics (2 pretransition and 2 posttransition) were enrolled in a 6-week study to test the DigiBete Chatbot. During the study, YP/YA completed web-based, validated, and standardized questionnaires at baseline, 2 weeks, and 6 weeks to evaluate quality of life and anxiety and depression, along with chatbot usability and acceptability. Qualitative interviews involving YP/YA, parents, and health care professionals explored their views on the chatbot. Data were analyzed using descriptive statistics and framework analysis.

**Results:**

Eighteen YP/YA were enrolled. Qualitative interviews were conducted with 4 parents, 24 health care professionals, and 12 YP/YA. Questionnaire outputs and the emergent qualitative themes (living with T1DM, using the chatbot, and refining the chatbot) indicated that the measures are feasible to use and the chatbot is acceptable and functional. In addition, responses indicated that, with refinements that incorporate the feasibility results, the chatbot could beneficially support YP/YA during transition. Users scored the chatbot as “good” to “excellent” for being engaging, informative, and aesthetically pleasing, and they stated that they would use it again. The results suggest that, with some adaptations based on user feedback, the chatbot was feasible and acceptable among the YP/YA who enjoyed using it. Our reactive conversational agent offers content (messaging and additional multimedia resources) that is relevant for the target population and clinically approved. The DigiBete Chatbot addresses the identified lack of personalized and supported self-management tools available for 11‐24 year olds with T1DM and other chronic conditions.

**Conclusions:**

These results warrant chatbot refinement and further investigation in a full trial to augment it prior to its wider clinical use. Our research design and methodology could also be transferred to using chatbots for other long-term conditions. On the premise of this feasibility study, the plan is to rebuild the DigiBete Chatbot to meet identified user needs and preferences and progress to a national cohort study to assess the usability, feasibility, and acceptability of a modified chatbot, with a view to proceeding to rollout for national and international use on the established DigiBete platform.

## Introduction

### Background

Type 1 diabetes mellitus (T1DM) is a serious chronic condition that affects 1 in 700 children and young people worldwide, with around 29,000 young people in the United Kingdom living with T1DM. Diabetes care is a priority in the UK National Health Service (NHS) Long Term Plan, reinforcing the need to improve the quality of care for children and young people with T1DM through bespoke quality improvement [1,2].

### Self-Management Problems in Young People and Young Adults With T1DM as They Transition

Transition (the purposeful and planned process of supporting young people and young adults [YP/YA] with T1DM to move from child to adult services) is poorly developed in many regions. There is a major gap in knowledge about transition readiness among YP/YA with chronic conditions.

YP/YA with T1DM are expected to learn about and perform multiple clinical self-management tasks while integrating self-management with daily activities. One-third of YP with T1DM show evidence of early diabetes-related complications by the time of transition, and they have a 2.5-fold elevated risk of poor glycemic control by the time of their first adult health service visit. Blood glucose control substantially declines among those aged 18-30 years, with only 14% meeting the required level. Critically, poorly controlled T1DM can cause acute life-threatening complications such as diabetic ketoacidosis and disabling chronic complications, including both microvascular and macrovascular disease [3].

Following transition, YA can experience significant deterioration in diabetes control, increased anxiety levels, and reduced quality of life [4-9], all of which challenge the YP/YA and their families and put a burden on the NHS [10].

### Inadequacy of Existing Support Measures

The highest rates of diabetic ketoacidosis are seen in individuals 15-20 years of age [11] (the age when they are transitioning between NHS pediatric and adult services); this is suggestive of transition support measures not reaching all YP/YA. During standard care, when YP/YA are transitioning, they typically receive support from both pediatric and adult teams to ease the process. However, interim support is often inconsistent, and clear information about the transition remains difficult to access. Clear, accessible resources could improve this process, helping YP/YA navigate the process with confidence and ensuring they receive the support they need at every stage.

A systematic review in 2022 demonstrated that adequate support for YP throughout transition results in improved glycemic control, improved clinic attendance, fewer episodes of hospitalization, lower rates of hypoglycaemia, better self-management, and increased knowledge of T1DM [12]. A more recent scoping review [13] found that a combination of self-management support digital health technologies (DHTs) exists, although no supportive chatbots with components to help YP/YA with T1DM were identified [13].

### Patient and Public Involvement in This Study

Patient and public involvement was central to our study design and delivery. Members of the preexisting DigiBete Expert User Group (EUG) who are YP/YA with T1DM had informed development of the DigiBete platform and app and advised on this study. For example, EUG members guided development of recruitment materials and processes; the content and design of initial chatbot resources; and dissemination. To avoid any conflict of interest, EUG members were not eligible to participate in this study.

The purpose of this paper is to build on our recent scoping review [13] and present the results of a feasibility study that addressed two objectives:

1. To evaluate DigiBete Chatbot, the first user-led, developmentally and age-appropriate, clinically approved transition chatbot for 11- to 24-year-olds with T1DM including underserved, seldom heard, and vulnerable groups.
2. To assess the feasibility and acceptability of a future study of the DigiBete Chatbot in terms of recruitment, retention, data collection procedures, and performance of study measures in this population.

## Methods

### Overview

We conducted a prospective, multimethod, nonrandomized feasibility and acceptability study to enable YP/YA, health care professionals (HCPs), and parents to access the DigiBete Chatbot and associated online materials and to allow researchers to collect feedback from participants through validated questionnaires and qualitative interviews. As this was a feasibility study, a power calculation was not required, and significance was not calculated because of the small sample size [14].

### The DigiBete Chatbot Intervention

This feasibility study forms part of a phased approach to the development and evaluation of the novel DigiBete Chatbot, a complex intervention [15] for YP/YA with T1DM. DigiBete, an existing video platform and app, provides support for diabetes management in YP/YA. It was founded and is run by families living with T1DM, and it is a social enterprise funded by NHS England, with all content clinically approved [16].

The DigiBete Chatbot prototype was co-designed and developed in collaboration with YP/YA, parents, and HCPs in 4 NHS hospital diabetes services in England (2 pretransition and 2 posttransition). All content was quality assured by a consultant pediatrician who specializes in type 1 diabetes in YP/YA.

Based on the UK National Institute for Health and Care Excellence Evidence Standards Framework for DHTs, the DigiBete Chatbot is set at National Institute for Health and Care Excellence DHT tier 2 (level 1) and tier 3a (level 2) requirements. To our knowledge, this is the first chatbot to have been rigorously co-designed and evaluated with YP/YA with T1DM. The chatbot content is influenced by self-management theory, the COM-B (capability, opportunity, motivation-behavior change) approach, and the associated behavior change wheel [9,10,17-19]. The DigiBete Chatbot supports users through reactive messaging, providing links to sources of information on T1DM, and including content for peers and HCPs. The chatbot addresses a range of topics that acknowledge barriers and enablers to self-management, offering guidance on how to navigate life while developing physically and emotionally and becoming increasingly independent.

The chatbot used response logic to interpret the user input and generate suitable responses. This involved using natural language processing so that the chatbot could understand the user’s intent and provide the relevant information or resources to satisfy the user’s queries. In the initial stages of the chatbot build, intents (n=142), which are purposes or goals that are expressed in a user’s input, and entities (n=75), which are terms or objects that are relevant to a user’s intent and provide context for that intent, as well as questions, which were phrased in a variety of ways, were loaded into the chatbot. When the users inputted a query, based on the question, intent, and entities used, the chatbot would then choose the correct dialog flow, of which 136 were created, and surface the requested answer, which could be in the form of text, film, or a downloadable PDF.

The safety of the users is always a priority, and the language used by the chatbot was of equal importance. With safety in mind, the chatbot was preloaded with clinically approved, pedestrianized responses to questions and queries, which were necessary to ensure the user’s safety while using the chatbot. The scope of the chatbot was limited to the resources on the website only [16], to ensure the clinical accuracy and safety of the resources that the chatbot would surface for the user.

Initially, the EUG was tasked with inputting as many questions as they could think of to do with transitioning to adult services, and this served as the baseline for the information that the chatbot would need to present when queried. This immediately highlighted gaps in the resources provided by DigiBete in the transition age category, which resulted in a development sprint to create and adapt resources that could adequately fill the resource gaps. As more testing was carried out with the EUG and HCPs, the need for more content became greater and so more tailored content was created and adapted to cater to these information needs. A flowchart in Multimedia Appendix 1 details user interaction paths, providing an overview of this aspect of the user experience. A more detailed explanation of the DigiBete Chatbot initial development and refinement processes will be reported in a forthcoming publication.

### Recruitment

In the 4 participating NHS diabetes services (2 pediatric and 2 adult centers), those eligible to participate were YP/YA aged 11‐24 years, parents of participating YP, and all HCPs in the clinical multidisciplinary teams. Clinical teams, under the supervision of local principal investigators, used patient databases to identify eligible patients and requested permission to refer them to the researcher. Thrive by Design (a collective of specialist service designers and researchers who ensure inclusivity in NHS research by engaging underserved, seldom heard, and vulnerable groups) and the YP/YA from the EUG also supported the development of recruitment and data collection processes. In addition, they guided the development and design of initial chatbot resources and dissemination. YPs and YAs were initially approached by HCPs at the site where they were receiving treatment. The HCPs collected the contact details of those who expressed interest in the study. Researchers then followed up with these individuals to provide more detailed information and to obtain their consent and assent.

Researchers emailed the developmentally and age-appropriate study invitation letter and participant information sheet to interested participants and, where appropriate, their parents. These documents explained the purpose of the study, who the investigator was, the timing of the web-based survey, that they would be invited to participate in a semistructured interview at the end of their 6-week chatbot trial, and which data were stored and where and for how long. Where interest was indicated by individuals approached, the researcher then arranged to speak to them via teleconference or by telephone to answer queries and explain the process for providing digital (adult) informed consent (or assent for young people <16 years).

The sample was recruited using a combination of purposive, theoretical, and convenience sampling; we aimed for a total sample of 32‐40 YP/YA across the 4 study sites, but due to pragmatic constraints, including the prevailing COVID-19 pandemic and associated restrictions, a sample of 18 YP/YA (12 female) was recruited.

By incorporating multiple urban locations with distinct demographic profiles and involving local health care staff in recruitment, the strategy increased the likelihood of obtaining a diverse and representative sample of YPs and YAs across ethnic, socioeconomic, and geographic lines, thereby enhancing the credibility and inclusiveness of the study findings.

Recruitment commenced May 2023 and follow-up ended February 2024.

Before accessing the DigiBete Chatbot, participating YP/YA were contacted by a DigiBete developer (who was not a member of the evaluation team and who monitored DigiBete Chatbot usage) to explain the process of accessing and navigating the chatbot. Participating YP/YA received password-protected access to the chatbot for 6 weeks to allow them to ask questions, and they were encouraged to use it as often as they wished. To enable this, at the point of consent/assent, the researchers collected participants’ demographic details, including the email address that the YP/YA had previously provided when they initially registered for access to the DigiBete web platform and app. From their secure NHS email addresses, the researchers then forwarded the YP/YAs’ emails to the DigiBete team, who confirmed that the email address was consistent with the one held on record by DigiBete and arranged for the participant to have special access to the chatbot that appeared on their own DigiBete app for the duration of the study.

### Data Collection

#### Measures

At baseline, we collected demographic data from the YP/YA (age, sex, postal code, and ethnicity). The following measures were administered electronically to the participating YP/YA: the user version of the Mobile Application Rating Scale (uMARS) [20], the Hospital Anxiety and Depression Scale (HADS) [21], the 36-Item Short Form Survey Instrument (SF-36) [22], and a modified version of the System Usability Scale (SUS) [23-25]. The measures were administered at 3 time points: before using the chatbot (time zero, T0), 2 weeks later (time 1, T1), and 6 weeks after first using the chatbot (time 2, T2). All questionnaires were imported into Qualtrics^XM^ software, and a secure database was created for data collection and analysis. The usability and technical functionality of the electronic survey had been tested by YP/YA advisors before fielding the questionnaires. The researchers allocated each study participant with a unique, anonymized study number/identifier and YP/YA participants were reminded of this at every communication during the chatbot evaluation. YP/YA also received email messages linking them to the questionnaire database; they accessed the database via their unique identifier, then entered the relevant study time point (T0, T1, T2) and received access to the correct questionnaires for that time point. Noncompleters received 1 reminder after 2 weeks. Multimedia Appendix 2 shows details about the measures used.

#### Qualitative Interviews and Focus Groups

After T2, YP/YA were invited to participate in a qualitative interview to ascertain their views on the DigiBete Chatbot’s design, content, and usability. In addition, parents of participating YP were invited to participate in qualitative interviews to explore their views on the chatbot’s potential to enable their child to become autonomous in self-management, and focus groups were conducted with HCPs to determine their views on the chatbot’s content and their role in supporting the chatbot if it was later deemed suitable to became part of standard care delivery. During interviews/focus groups with parents and HCPs, the chatbot was demonstrated by the researchers, and participants were invited to suggest questions (which the researcher typed in) and discuss the answers generated. Interviews and focus groups were conducted via teleconference or in person in a quiet room in the hospital by researchers trained in these methods.

### Data Analysis

#### Quantitative Analysis

Data were analyzed using Qualtrics^XM^ software. Scores on the outcome measures were calculated and missing values on items were handled according to the methods prescribed by the developers. Consistent with the nature of the study and the small sample size, our postintervention analyses should be interpreted conservatively.

#### Qualitative Analysis

Qualitative data were analyzed using the Framework technique supported by NVivo [26], a recognized, systematic method for handling large amounts of qualitative data. Framework sits in a thematic methodology that is systematic, thorough, and grounded in the data but also flexible and enables easy retrieval of data to show others, thereby providing a clear audit trail. A rigorous, matrix-based method, it allows movement back and forth between levels of abstraction without losing the meaning of the “raw” data. Key quotations were labeled and identified for later retrieval and reporting. In addition, Framework allows both between- and within-case analysis and involves a process of familiarization with the data, identification of themes, indexing, charting, mapping, and interpretation. In line with the inductive nature of qualitative research, themes derived during qualitative data collection and analysis supplemented interview topics with new lines of inquiry [26]. Interview recordings were transcribed by a university-approved transcriber. A sample of anonymized transcripts was independently reviewed by 2 researchers and then discussed until a consensus was reached to assess interrater reliability and strengthen trustworthiness.

### Ethical Considerations

The NHS Research Ethics Committee, the Integrated Research Application System (IRAS reference 292053), the Lead NHS Trust Research and Innovation Department, and the Ethics Committee at the lead/corresponding author’s university approved this study. In line with Research Ethics Committee approval, we did not ask those who declined or dropped out for a reason. Data were collected and retained in accordance with the Data Protection Act 2018. Consent/assent forms and investigator site files were electronic. All data were managed in accordance with the data management plan of the lead/corresponding author’s university. Encrypted audio recordings and transcripts of qualitative interviews/focus groups and questionnaire data have been stored in password-protected files on a university server and will be retained for 5 years. The raw data were only shared with researchers working on the relevant work packages. Electronic data were transferred using encrypted devices according to standard university data-protection policies. No personally identifiable information was used in the reporting. After study completion, participants received a £25 (USD 34) online shopping voucher to thank them for their time.

## Results

### Overview

**Figure 1.**
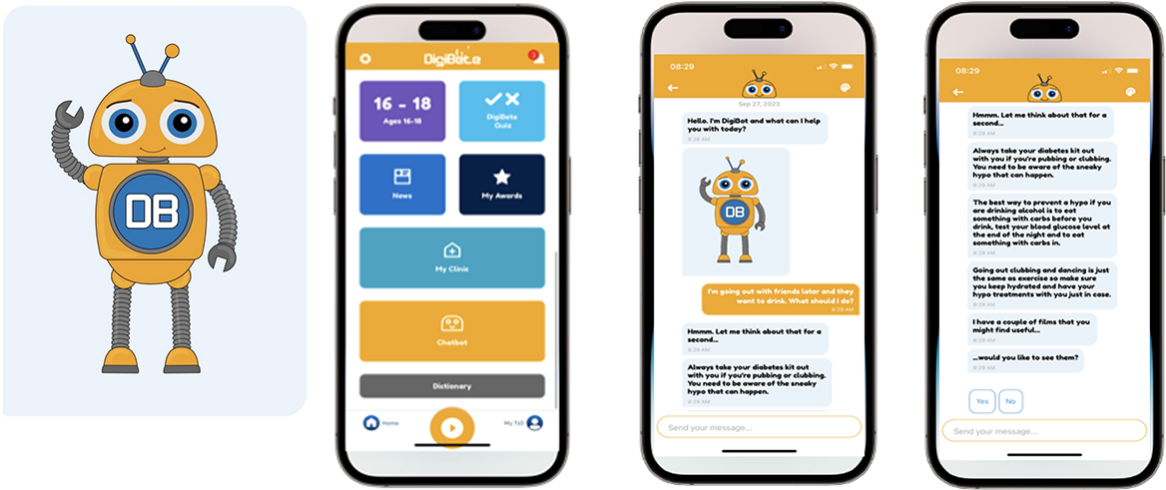
Screenshots of the DigiBete Chatbot used in the feasibility study. Chatbot image as viewed by users.

Figure 1 illustrates screenshot examples from the chatbot as viewed by participants.

Our a priori feasibility criteria rates were partially met. We aimed to recruit 32‐40 YP/YA but due to pragmatic constraints during the COVID-19 pandemic, we recruited only eighteen 11‐ to 24-year-olds with T1DM (12 female; 16 British, 2 White European). Participants’ engagement with study procedures varied. The mean age was 17 (SD 4.52) years. The mean number of years since diagnosis was 7.94 (SD 5.57). Three of those who consented/assented did not participate; one completed all questionnaires but not the interview, and another two did not complete questionnaires at the final time point (T2) and did not participate in the interview. Overall, 15 (72% of the participants) fully completed the feasibility study, 11% (n=2) partially completed it, and 17% (n=3) did not engage. Of the 15 who completed measures and interviews, the mean age was 17 (SD 4.52) years, the median age was 16 years, and the mean number of years since diagnosis was 7.94.

Twenty-five HCPs and 4 parents participated in individual or group interviews (Multimedia Appendix 3 for more details about participant characteristics). Semistructured interviews (n=16) were conducted online and focus groups (n=4) were conducted online or in person in a quiet room at the hospital, at times convenient for the participants, by experienced qualitative researchers who facilitated discussions based on topic guides. Discussions were digitally recorded, and transcripts were anonymized and coded by the researchers.

### Quantitative Results

Table 1 shows the 3 time points for administration of questionnaires, and the number of YA/YP who assented/consented and completed the questionnaires.

**Table 1.** Questionnaire completion time points and number of completions.

Questionnaires	T0 (baseline)	T1 (2 weeks later)	T2 (6 weeks later)
Hospital Anxiety and Depression Scale	N=15	N=15	N=14
36-Item Short Form Survey Instrument	N=15	N=15	N=14
System Usability Scale	N/A^a^	N=15	N=14
User version of the Mobile Application Rating Scale	N/A	N/A	N=14

a N/A: not applicable.

In the following subsections, quantitative results are labeled according to the focus of each questionnaire.

### Usability

The SUS was used to evaluate the usability of the chatbot, addressing 3 key aspects of usability: effectiveness, efficiency, and satisfaction.

SUS is a 10-item questionnaire with 5 response options for each item (from Strongly Disagree to Strongly Agree). The scores were calculated using the method recommended by Hägglund and Scandurra (2021) [27].

YPs and YAs were required to answer all SUS items. As reported in Table 2, each item was scored from 0 to 4 (with 4 being the most positive response). Scoring SUS involved two initial steps:

1. For odd-numbered positive statements: subtract 1 from the user response.
2. For even-numbered negative statements: subtract the user responses from 5.

**Table 2.** Mean System Usability Scale scores of the DigiBete Chatbot at time points T1 (2 weeks of use) and T2 (6 weeks of use).^a^

Item	Modified System Usability Scale statement	T1 (15 participants)	T2 (14 participants)
1	I think that I would like to use the chatbot frequently	2.1	2.3
2	I found the chatbot unnecessarily complex	3.4	3.6
3	I thought the chatbot was easy to use	3.5	3.7
4	I would need the support of a technical person	3.9	3.9
5	I found the various functions in the chatbot were well integrated	2.6	3.1
6	I thought there was too much inconsistency in the chatbot	2.9	2.4
7	Most people would learn to use this system very quickly	3.7	3.9
8	I found the system very cumbersome to use	3.3	3.4
9	I felt very confident using the chatbot	2.9	3.5
10	I needed to learn a lot of things before I could get going with the chatbot	3.9	3.9

a In T1, there were 6 items scored above 3.0. In T2, there were 8 items scored above 3.0.

Table 2 shows that two calculations were performed at T1 and T2: (1) mean value of all individual answers and (2) number of items scored above 3.0.

Most items had a mean score above 3.0. At T1, six items scored above the mean of 3.0, whereas at T2, eight items scored above the mean of 3.0.

This shows a positive trend: more items were rated positively at T2 than at T1, suggesting an overall improvement in user-perceived usability, which is consistent with the increase in the overall SUS score (discussed later in this section). However, there was a reduction in the score for item 6 in relation to the consistency of the responses given by the chatbot. This could indicate a specific usability issue; even though overall usability improved, users felt the chatbot became less consistent in its responses.

There was no change in the scores for items 4 and 10. No change implies these aspects (perceived need for support and learning burden) remained stable—neither improved nor worsened.

To calculate the mean and median of all scores, the scores for each item were then added together and multiplied by 2.5 to obtain the final score. The final score ranged from 0 to 100, with higher scores indicating better usability.

At T1, the mean was 80.5 and the median 82.5; at T2, the mean was 85.2 and the median 86.3. The results can be interpreted using Lewis and Sauro’s interpretation [23] where:

- An SUS score of 80.5 (at T1) was designated as good or an A– grade.
- An SUS score of 85.2 (at T2) was designated as the best imaginable or an A+ grade.

These high usability scores indicate that the YPs and YAs found the chatbot easy and pleasant to use, and that usability improved from T1 to T2.

### Quality

The uMARS was used to assess the quality of the chatbot. All questions were mandatory for YPs and YAs to answer.

Table 3 provides a detailed breakdown of the number of questions in each category and the highest scores obtained in each category. Based on YP/YAs’ responses, the mean score and mean percentage were calculated.

**Table 3.** Mobile Application Rating Scale results by category for the DigiBete Chatbot.

Category	Number of questions	Highest total score that can be obtained	Mean score for each category	Percentage of the mean score
Engagement	5	25	17.43	69.7
Functionality	4	20	17.86	89.3
Aesthetics	3	15	12.93	86.2
Information	4	20	15.92	79.6
App subjective quality	4	20	12.79	64
Perceived impact	6	30	19.79	66

There are 4 uMARS categories that indicate the quality aspect of the chatbot: Engagement, Functionality, Aesthetics, and Information. Functionality was the highest-rated category (89%), indicating YPs/YAs found the chatbot easy to use, stable, and technically sound. This is a major strength. Aesthetics (86%) was also very high. YP/YAs found the chatbot visually pleasing and well-designed. Combined with high functionality, this suggests good user experience design. The information (80%) category means the chatbot provides trustworthy and relevant content, though there may still be room for refinement. However, the lowest category for quality was Engagement (70%), which could indicate that the chatbot could be more interactive, interesting, or customizable.

Table 4 shows responses (5-point Likert scale plus an N/A response option) for the Engagement, Functionality, Aesthetics, and Information items. Each uMARS item is rated on a 5-point Likert scale from 1 (inadequate) to 5 (excellent). All the quality categories (Engagement, Functionality, Aesthetics, and Information) were mostly rated 4 (good) or 5 (excellent).

**Table 4.** Mode of participants’ responses for the user version of the Mobile Application Rating Scale.

Categories	1 (inadequate)	2	3	4	5 (excellent)	N/A^a^	Total responses
Engagement: entertainment, interest, customization, interactivity, target group	4	7	19	31	9	0	70
Functionality: performance, ease of use, navigation, gestural design	0	1	1	25	29	0	56
Aesthetics: layout, graphics, visual appeal	0	0	4	21	17	0	42
Information: quality and quantity of information, visual information, credibility of source	0	3	10	22	19	2	54

a N/A: not applicable.

However, 2 respondents selected the N/A response option in the Information category, indicating that they received no information regarding the quality and quantity of the content, availability of visuals, and credibility of the sources contained in the chatbot to answer their questions. These results indicate that although over 50% of YPs and YAs found the DigiBete Chatbot easy to navigate and use, it needs further modifications to make it more accessible for some users.

Table 5 lists the subjective quality item ratings; 86% of the respondents would potentially recommend the chatbot, and 14% would recommend it. However, more than half (64%) would not pay for the DigiBete Chatbot, although over 50% rated it as 4- or 5-star.

**Table 5.** Quality of the chatbot (subjective items).

Subjective chatbot quality items from the user version of the Mobile App Rating Scale	Value (N=14), n (%)
**Would you recommend the DigiBete Chatbot to people who might benefit from it?**	
1: Not at all	0 (0)
2	0 (0)
3: Maybe	6 (43)
4	6 (43)
5: Definitely	2 (14)
**How many times do you think you would use DigiBete Chatbot in the next 12 months if it was relevant to you?**	
1: None	0 (0)
2: 1‐2	0 (0)
3: 3‐10	7 (50)
4: 10‐50	6 (43)
5: >50	1 (7)
**Would you pay for the DigiBete Chatbot?**	
1: Definitely not	9 (64)
2:	2 (14)
3:	3 (21)
4:	0 (0)
5: Definitely yes	0 (0)
**What is your overall (star) rating of the DigiBete Chatbot?**	
1: One of the worst apps I have used	0 (0)
2:	0 (0)
3: Average	6 (43)
4:	6 (43)
5: One of the best apps I have used	2 (14)

### Anxiety and Depression

The HADS was used to measure symptoms of anxiety and depression.

The HADS results showed that anxiety subscale values were higher than those of the depression subscale (Table 6). Specifically, some YPs/YAs reported a borderline or abnormal anxiety score (higher mean for anxiety), whereas their depression scores were within the normal range. The anxiety scale also had higher maximum values, which were within the abnormal range. One participant had an anxiety score close to the maximum abnormal range (values of 19 and 20).

**Table 6.** Proportion of participants with normal, borderline, and abnormal levels of depression and anxiety according to Hospital Anxiety and Depression Scale scores.

	T0 (N=15)		T1 (N=15)		T2 (N=14)	
	Depression	Anxiety	Depression	Anxiety	Depression	Anxiety
Normal	93.33	53.33	93.33	60.00	92.86	64.29
Borderline	0.00	20.00	0.00	13.33	7.14	0.00
Abnormal	6.67	26.67	6.67	26.67	0.00	35.71

Most reported a normal range of depression scores at all time points. However, between the anxiety and depression subscales, anxiety had a higher percentage at the time points: 26.67% at T0 and T1 and 35.71% at T2. Only 6.67% were classed as “abnormally” depressed at T0 and T1, while no participant was considered “abnormally” depressed at T2.

This indicates that anxiety was more prevalent than depression, where more participants had borderline or abnormal anxiety, and extreme anxiety was observed in isolated cases (at least one participant had very high anxiety scores, near the maximum abnormal threshold of 21).

Depression was largely not a concern because most remained in the normal range for depression at all time points.

### Health Status and Quality of Life

The SF-36 was used to assess overall health status and quality of life across physical, mental, and social domains.

YP/YA were required to answer all 36 questions at all time points. Table 7 shows the average and SD of YP/YA scores across the 3 time points for each health domain. The Physical Functioning domain yielded consistently high mean scores with no significant change observed between the time points. This could indicate that the YPs and YAs can perform basic activities (eg, walking) and instrumental activities of daily living (eg, bathing and dressing). Items in this domain also include participants’ perceived ability to perform vigorous activities, such as running and lifting heavy objects, and moderate activities, such as moving a table and bowling.

**Table 7.** Quality of life results from the 36-Item Short Form Survey Instrument at 3 time points.

Domain	T0 (N=15), mean (SD)	T1 (N=15), mean (SD)	T2 (N=14), mean (SD)
Physical Functioning	92.33 (9.42)	92 (8.62)	91.43 (12.16)
Role limitations due to Physical health	80.00 (30.18)	88.33 (20.85)	83.93 (33.41)
Role limitations due to Emotional health	77.78 (32.53)	80.00 (37.37)	80.95 (33.88)
Energy/Fatigue	61.67 (24.03)	62.00 (19.71)	56.79 (23.66)
Emotional Well-being	69.07 (19.85)	70.13 (20.50)	68.86 (23.70)
Social Functioning	85.00 (23.24)	82.50 (24.91)	86.61 (24.25)
Bodily Pain	83 (15.12)	83.50 (15.26)	83.93 (16.60)
General Health	59.33 (15.91)	63.00 (13.73)	65.36 (16.69)

The domain Energy/Fatigue, however, has 2 mean values, which are the lowest in comparison to the mean values in other domains. In this domain, questions related to emotional well-being (feeling energetic, lively, and enthusiastic) and physical well-being (feeling worn out and tired). Low scores in this domain could indicate that they are feeling drained and demotivated.

Overall, there was no significant change across the 3 time points for all domains.

### Qualitative Findings

#### Overview

A total of 41 participants (6 YP aged 11‐15 y, 6 YA aged 16‐24 y, 4 parents, and 25 HCPs) participated in an individual or focus group interview. Using framework analysis, themes derived during qualitative data collection and analysis (living with T1DM, using the DigiBete Chatbot, and refining the DigiBete Chatbot) supplemented interview topics with new lines of inquiry [26,28]. The full framework of themes/subthemes derived from qualitative data analysis is available in Multimedia Appendix 4. Below, we present summary narratives juxtaposed with verbatim quotations to illustrate the derived themes.

#### Living With T1DM

YP and YA spoke of the shock of diagnosis and the overwhelming information related to it, as one YA described:

I think my parents took it harder than I did, and no one really knew about it. Dad struggled …. Lockdown [Covid] difficult straight after diagnosis.

Also, her parents thought the diagnosis was wrong at first, so when it was confirmed correct:

…that was quite sad. But after that, once I’d accepted it, found it quite insightful. It was hard, get used to it, [but] becomes second nature.

[YA aged 22 y, diagnosed aged 19 y]

YP/YA acted as peer supporters and educators for those around them and were aware that learning more about their condition may be stressful for others; they generally accepted living with T1DM and could not remember a time before diagnosis, although some indicated frustration about the inconvenience of self-management.

Parents showed an awareness of future needs as their child moved toward independence, and they were pleased with how their child coped:

He’s done very well to adapt to it.

[Parent of 13 year old, diagnosed at 9 y]

#### Using the DigiBete Chatbot

The participants were positive about the chatbot and its benefits to users and those around them. It provides reassurance, generally offers accurate advice, and has potential as an educational tool for others. A short, easy-to-read format was preferred, and all found it straightforward to use. A strong preference was indicated by YPs/YAs and HCPs for short text messages and videos over links to websites and PDFs. Some participants indicated that the chatbot was not their only source of T1DM information and reassurance and that they may not rely on it in an emergency.

One YP thought the chatbot might be useful when he is out with friends, and while he seemed confident about managing his T1DM, he thought he may in future need further advice from the chatbot with self-management, for example to calculate alcohol units in a specific drink:

Yeah because, like drinking alcohol maybe?

[11-year-old boy, diagnosed at 2 y of age]

Without exception, YP, YA, and parents would recommend the DigiBete Chatbot to others because they liked its accessibility, together with the reassurance and knowledge it provides. Generally, HCPs received the chatbot positively and thought it might fill a gap by addressing questions typically not asked in the clinic by YP/YA. As one HCP said, patients may ask, for example:

*‘What happens if I give too much insulin?’* or *‘Can I lose weight by not taking insulin?’. It’s those unsaid things [often] not asked in clinic*.

[Clinical psychologist]

However, some HCPs were more reserved about recommending it in its current stage of development, citing safety and accuracy concerns. For example, some were concerned about the accuracy of some information provided, given the wide age range of prospective users. Some HCPs were also worried that some terminology used by the YP/YA when asking questions of the chatbot may be interpreted incorrectly. One HCP said they would:

…like to see safety mechanisms built in before recommending its use…

[Consultant pediatrician]

#### Refining the DigiBete Chatbot

The appearance of the chatbot was well received by participants, although options for customization of the avatar were suggested (this finding corresponds with results from the uMARS). Participants recommended further refinement of the accuracy of chatbot responses, with options to tailor or streamline responses by age, comprehension abilities, language preference, and voice activation. These recommendations are consistent with the results of the uMARS, which also indicate that the chatbot needs further modification, with improvements to enable users to customize it and make it more entertaining. See Multimedia Appendix 5 for participants’ (YP/YA, parents, and HCPs) specific qualitative suggestions for DigiBete Chatbot improvements and the developers’ responses regarding the feasibility of integrating these suggestions in a future version.

The complexity of the chatbot’s responses could be reduced to encourage engagement with the information provided. For example, some highlighted a need for the chatbot to supply short, text-based answers first, rather than what they sometimes received in response to their questions (ie, lengthy and complicated information documents). Participants suggested that safety issues around mental health and emotional support should be enhanced by referencing correct and appropriate support in a timely manner. Suggestions for expanding chatbot content focused on the provision of reassurance and accurate contemporary information while recognizing users’ developmental stages and the potential for experimentation alongside peers. For instance, one diabetes nurse was concerned that those with “English as a second language may struggle” and that YP/YA may think they are talking directly with their clinical team via the chatbot; this respondent also highlighted a need to be clear that the diabetes team are not available 24/7, so they:

…may need DigiBete Chatbot to remind them in [a] bold, big message flashing up and give contact numbers.

This diabetes nurse also said it would be helpful if information about meals and carbohydrates in food outlet chains was linked to the chatbot, as YP/YA may be embarrassed to request this information when eating out.

The chatbot could encourage compliance with medications, clinic attendance, and/or engagement with specialist teams. The difficulty of deciding what information to include in the chatbot was acknowledged, along with the extensive age range of potential users.

### DigiBete Chatbot Engagement Metrics

Over the course of the feasibility study, 65 conversations of varying lengths were recorded, with an average of 3.3 interactions per conversation. The user conversations were captured and exported from the chatbot to analyze how it was performing. The trial data were anonymized, so we are unable to identify who the users were. Within IBM Watson x Assistant, we collected the conversations and determined how many interactions in each conversation involved the chatbot; sometimes only one question was needed for the user to find something out quickly, but in most conversations, multiple questions were asked.

## Discussion

### Principal Findings

To our knowledge, this is the first national (United Kingdom/NHS) or international self-management chatbot to be co-designed, developed, and evaluated by patients living with T1DM during their transition journey, as well as by their parents and HCPs [1,3-13,29]. The results reported here will inform the design and delivery of a future large-scale cohort study to assess the acceptability, functionality, and usability of the DigiBete Chatbot when assessed by 11‐24 year olds with T1DM. Even with the small sample size of this nonrandomized feasibility and acceptability study and no other transition chatbots to compare DigiBete with, the results suggest that with some adaptations based on user feedback, the chatbot is feasible and acceptable among the YP/YA who enjoyed using it. This and the proposed cohort study will pave the way for the development of a national and fully functional NHS-approved, developmentally and age-appropriate, online and app-based chatbot to “go live” after the end of the cohort study.

The DigiBete Chatbot addresses the identified lack of personalized and supported self-management tools available for 11‐24 year olds with T1DM and other chronic conditions [9,13,30,31]. Our reactive conversational agent offers content (messaging and additional multimedia resources) that is relevant for the target population and clinically approved. The chatbot was co-designed and co-developed with and for YP/YA with T1DM as they transition from child to adult health services, providing them with informational support to enhance their knowledge, skills, and confidence (as they transition toward adulthood and independent self-management) in navigating their T1DM self-management journey [9,18]. Scores from the SUS, designed to measure users’ perception of the usability of a system, indicated improvement from baseline to the second time point, with improvements in perceived ease of use, functionality, and confidence. This demonstrates that participants viewed the chatbot as highly usable. Having used the chatbot for 6 weeks, YA/YP who were more likely to use it in the future found that its functions were well integrated, became more confident in using it, and perceived the chatbot to operate in a consistent way. This aligns with the consolidated star rating from the uMARS showing that more than 50% of participants allocated the DigiBete Chatbot 4 or 5 stars out of 5, with no respondents rating the app below 3 out of 5 stars.

After using the chatbot, participants scored it highly in the domains of engagement, functionality, aesthetics, and the information provided, with lower overall scores in subjective quality and perceived impact. Functionality scored highly, suggesting that the chatbot met the intended needs of users in relation to performance, ease of use, navigation, and gestural design. Despite this, feedback from the HADS questionnaire demonstrates that the chatbot is unlikely to have a significant impact on depression and anxiety over the short time scale applied to this feasibility study. This aligns with the lower score for the perceived impact in the uMARS questionnaire. Similarly, from the SF-36 results, the chatbot did not impact physical or emotional well-being and did not improve social functioning, although there was a trend toward an improvement in general health.

Qualitative data demonstrate that participants were positive about the chatbot and its benefits to users and those around them. In the qualitative assessment, users recognized that the chatbot could identify issues around mental health and emotional well-being and suggested that “safety-netting” within the chatbot could support timely referral and so was required. Responses in uMARS also demonstrated that the chatbot was aesthetically pleasing to users, although in future developments, participants would like the ability to customize the chatbot based upon their personal preferences. Reassuringly, all participants would recommend the chatbot and would use it again during the following 12 months.

The DigiBete Chatbot provides reassurance and gives accurate advice, and all participating YP/YA and parents would recommend it to others. HCPs also received the chatbot positively; however, more work was suggested by some HCPs to develop it further. Quantitative analysis also demonstrated that the chatbot was usable; over time, YP/YA gained confidence in its use. Users particularly liked the functionality and information provided. However, the areas of improvement cited include the need for more technical information and support when using the chatbot.

Before proceeding further, and based on qualitative comments from YP/YA, parents and HCPs recommended improving the usability of the DigiBete Chatbot by, for example, enabling it to remember each user at return visits, recording details relating to user characteristics to assist the persuasive conversational capacity of the intervention, accommodating user language choices in their conversational dialogue, and supporting customization and personalization (see Multimedia Appendix 5 for additional qualitative suggestions). Given the increasingly ubiquitous use of smartphones and popularity of chatbots in daily life for YP/YA, chatbots such as DigiBete may also be an increasingly popular and scalable solution to promote confident and competent self-management during transition by YP/YA living with other chronic conditions [8]. For example, YP/YA with juvenile arthritis or chronic kidney disease [32,33] also often experience high levels of self-management support needs before, during, and after transition to adult health care; these needs could be addressed by tailored, disease-specific chatbots such as the DigiBete Chatbot.

In the next phase of the DigiBete Chatbot development and evaluation, user safety will continue to be of the utmost importance. The current version is designed to meet the transition needs of YP/YA aged 11‐24 years. The next phase will open the chatbot up to other age groups so the language used by the DigiBete Chatbot will have more complexities due to this wider age and developmental differences; the vocabulary used by the different ages will vary as they query the chatbot. The resources within the revised DigiBete Chatbot will need to reflect the age and developmental stage of the person making the query, so again, language matters. The resources will all be housed on the DigiBete website, which will ensure clinical safety and accuracy. Although the resources will be clinically accurate and safe, the language used by the chatbot will be pedestrianized so that users do not feel that they are in a clinical environment. If a user’s query indicates signs of distress or suggests they are in crisis or an emergency, then the chatbot will respond by signposting other agencies that can help, along with advice to contact their local diabetes team for help and support.

### Strengths and Limitations

A nonrandomized preliminary feasibility study (as opposed to a pilot randomized controlled trial, which resembles an intended randomized controlled trial in aspects such as having a control group, randomization, and determining efficacy and effectiveness) effectively met our aims, which included an intention to determine access to participants (eg, willingness of clinicians to introduce eligible patients/parents to the study, participant responses to invitations, barriers to participation, and the feasibility and suitability of assessment procedures and outcome measures). Therefore, we regard this as a strength of our study design as it helped us to achieve these aims.

The study design was strengthened by basing it on the Template for Intervention Description and Replication (TIDieR) checklist (Checklist 1 details this as a guide) and using a multimethod design in which qualitative data helped to foster new insights into factors underpinning quantitative data.

The feasibility and acceptability testing enabled us to refine and put study procedures in place and incorporate inclusion and exclusion criteria and processes for tracking enrollment and data collection. The research design also enabled us to evaluate the performance of the measures used in combination with the qualitative findings to determine if the intervention was acceptable. These procedures were tested in a sample drawn from the target population for a future full-scale study. We were also able to train research staff in administering study procedures—including participant identification and referral, recruitment, enrollment, and data collection—and chatbot development staff in teaching participants how to use the chatbot.

The DigiBete Chatbot has the potential to beneficially affect patients’ self-reported self-management outcomes, and a full-scale study of its usability and acceptability following iterative changes to the chatbot based on the reported findings was found to be feasible. Most data were collected remotely, which was convenient for participants during the COVID-19 pandemic.

The study had some limitations; for example, while the primary source of participant identification and recruitment was clinical appointments, this process could be further strengthened in the future by clinicians inviting existing DigiBete users via the app. In addition, due to the prevailing COVID-19 restrictions at the point of recruitment, there was a relatively small sample size and limited diversity regarding digital exclusion and ethnic diversity. We did not analyze the characteristics of participants who engaged more versus less, which could have yielded valuable additional insights into acceptability and usability, and we did not collect exploratory data on clinical outcomes such as glycated hemoglobin to detect significance, as this was outside the scope of this study. There are also limitations to the reactive chatbot’s content/functionality and content recommendations; some of these recommendations cannot yet be achieved owing to the current technological capabilities of the chatbot. A future chatbot evaluation would be strengthened by addressing these limitations.

Although questionnaire completion rates were less than optimal, the measures could be used in a future full study with small amendments. For example, the respondent burden for the SF-36 was high as it involves 8 domains across 36 items, but this could be reduced to 17 items by selecting only the 3 most relevant domains: physical function (10 items), social function (2 items), and mental health (5 items).

The chatbot was only available to each user for a 6-week period; it is likely that this period was too short to demonstrate a significant impact on the overall physical, emotional, and social health of YP/YA. For instance, the study period may have been insufficient to produce significant changes in anxiety and depression, as meaningful improvements in mental health often require extended support, structured interventions, and time for individuals to process information and adapt to their new circumstances. Mental health progress is influenced by multiple factors, including the severity of symptoms, engagement with therapeutic techniques, and additional support systems. Although chatbots can provide immediate coping strategies, emotional validation, and psychoeducation, sustained improvements in mood and anxiety levels typically develop over months rather than weeks. Furthermore, given that this was a feasibility study, the small number of responses may have been insufficient to demonstrate an impact on emotional and physical well-being. In future studies of the DigiBete Chatbot, a longer trial period with more participants in more centers to assess impact in the domains of the HADS and SF-36 may provide a more realistic view of its impact.

Our participating YP/YA were predominantly female and experienced in the use of smartphones and chatbots. Geographic sample limitations included recruitment only from urban areas but no specific representation from rural areas where internet access may be less reliable. Because of the small sample size, the chatbot may have been trained on biased data, potentially leading to inadequate responses. The chatbot is designed to have a gradual or cumulative effect on users’ health behaviors and many health outcomes take time to manifest so such a short study may not have been able to capture this progression, especially because chatbots can sometimes feel cold and robotic to users, lacking the empathy and nuance of human interactions [34].

Potential biases may have affected the study outcomes. For example, although we worked with a collective of specialist service designers and researchers who aim to ensure inclusivity in NHS research by engaging underserved, seldom heard, and vulnerable groups and who supported recruitment and data collection, differences may exist between patients who volunteered and those who refused participation (self-selection bias). In addition, although we made every effort to accommodate participants’ commitments when scheduling qualitative interviews or focus groups, some may have been inadvertently excluded because of personal time constraints (participation bias). Finally, some YP/YA participants may have had difficulty recalling their thoughts about the chatbot when completing outcome measures at 2 weeks and 6 weeks and qualitative interviews at 6 weeks (recall bias). Despite controlling for these and other types of bias, unidentified limitations may still exist.

Because of the study limitations, the results should be interpreted with caution and will require validation in a larger study with a more representative sample and potential generalizability of results. Future research needs to determine whether our findings extend to a more heterogeneous sample.

### Conclusions

The impact of chatbots in T1DM care remains unclear. Prior to examining the DigiBete Chatbot’s effectiveness in the future, its usability, acceptability, and impact on users’ psychological well-being in the target YP/YA population were examined. The DigiBete Chatbot was deemed usable, acceptable, and feasible for delivery. Our results warrant some refinement of the chatbot content based on the recommendations reported here and further investigation prior to its wider use in clinical practice. Our research design and methodology could also be transferred to the development and evaluation of chatbots for YA/YP living with other chronic conditions before and after transition.

On the premise of this feasibility study, the plan is to rebuild the DigiBete Chatbot to meet identified user needs and preferences and progress to a national cohort study to assess the usability, feasibility, and acceptability of a modified chatbot, with a view to proceeding to roll it out for national/international use on the established DigiBete platform.

## Supplementary material

10.2196/74032 Multimedia Appendix 1 User interaction flowchart.

10.2196/74032 Multimedia Appendix 2 Details on measures used.

10.2196/74032 Multimedia Appendix 3 Participant characteristics.

10.2196/74032 Multimedia Appendix 4 Framework of derived themes from qualitative data.

10.2196/74032 Multimedia Appendix 5 Participants’ qualitative suggestions.

10.2196/74032 Checklist 1 TIDieR checklist.

## Abbreviations

DHT: digital health technologies
EUG: Expert User Group
HADS: Hospital Anxiety and Depression Scale
HCPs: health care professionals
NHS: National Health Service
SF-36: 36-Item Short Form Survey Instrument
SUS: System Usability Scale
T0: baseline
T1: time 1 (2 weeks)
T1DM: type 1 diabetes mellitus
T2: time 2 (6 weeks)
TIDieR: Template for Intervention Description and Replication
uMARS: user version of the Mobile Application Rating Scale
YP/YA: young people and young adults
