# Supplementary material for: DigiBete, a Novel Chatbot to Support Transition to Adult Care of Young People/Young Adults With Type 1 Diabetes Mellitus: Outcomes From a Prospective, Multimethod, Nonrandomized Feasibility and Acceptability Study
Source: JMIR Diabetes. 2025 Jul 23;10:e74032. doi: 10.2196/74032 (PMC12309419; doi:10.2196/74032)
Supplement: Multimedia Appendix 2 [file diabetes-v10-e74032-s002.docx]

**Multimedia Appendix Supplementary file 2 – Details about measures used**

**The System Usability Scale (SUS)** is designed to assess the usability and learnability of mobile apps. It is a 10-item questionnaire that is scored in a 5-point Likert scale [strongly disagree to strongly agree]. The SUS is widely used in studies on user centered design for mobile technologies, has been validated and has strong reliability in previous studies (*a*=0.70 to 0/91). The SUS used in this trial consisted of all 10 items that were slightly modified after piloting with young people to meet the identified needs and preferences of our target population (Table A). All SUS items were mandatory for participants to respond. In addition to the 10 items a free text response box was available.

Table A: The SUS statements and the study modified statements

| **Item** | **SUS statement** | **Modified SUS statement** |
| --- | --- | --- |
| 1 | I think that I would like to use this system frequently | I think that I would like to use the chatbot frequently |
| 2 | I found the system unnecessarily complex | I found the chatbot unnecessarily complex |
| 3 | I thought the system was easy to use | I thought the chatbot was easy to use |
| 4 | I think that I would need the support of a technical person to be able to use this system | I would need the support of a technical person |
| 5 | I found the various functions in this system were well integrated | I found the various functions in the chatbot were well integrated |
| 6 | I thought there was too much inconsistency in this system | I thought there was too much inconsistency in the chatbot |
| 7 | I would imagine that most people would learn to use this system very quickly | most people would learn to use this system very quickly |
| 8 | I found the system very cumbersome to use | I found the chatbot very cumbersome to use |
| 9 | I felt very confident using the system | I felt very confident using the chatbot |
| 10 | I needed to learn a lot of things before I could get going with this system | I needed to learn a lot of things before I could get going with the chatbot. |

**The (user) Mobile App Rating Scale (uMARS)** is a 23-item scale designed to assess engagement, functionality, aesthetics, informational quality and subjective quality of a mobile app. The developers later simplified MARS and piloted it with 13 YP to create the uMARS, a 20-item measure that includes four objective quality subscales: engagement, functionality, aesthetics, and information quality, and one subjective quality subscale (made up of four questions that measure a user's perception of an app: willingness to recommend the app, anticipated frequency of app usage, willingness to pay for the app, and overall rating). The uMARS was developed with 16–25-year-olds, after piloting uMARS informally with expert advisors aged 11 and 13 years, we concluded that it was too long, and some words were not easily understood by YP aged <16-years. With the developer’s permission we adapted it to a) alter some words and phraseology to make it more age- and developmentally appropriate and b) reduce the number of questions offered to participants.

NB In line with his request that: *the modified scale should not be made publicly available due to Queensland University of Technology’s IP restrictions,* we have not included our modified version of the scale that we used, in this manuscript.

**Hospital Anxiety and Depression Scale (HADS)** is a validated 14-item self-report measure, with seven items for the depression subscale and seven items for the anxiety subscale. Each item is rated using a four-point scale, with 0 to 3. 3 = higher symptom frequency. Total scores on each subscale = 0 to 21, normal (0–7), mild (8–10), moderate (11–14) severe (15–21). All HADS questions were mandatory for the participants to answer. The HADS has previously been used with eight- to 17-year-olds as well as adults. Completed HADS questionnaires were screened by the DigiBete chatbot study team soon after they were submitted by YP/YA to enable urgent referral of those reporting high scores or suicidal tendencies to appropriate mental health services.

**The Short-Form 36 (SF-36)** is a widely used and validated measure involving 36 items measuring eight dimensions of: physical functioning, role physical, bodily pain, general health, vitality, social functioning, role emotional, and mental health and an item that determines perceived differences in state of health over the past year. The SF 36 was previously administered to13 - 23-year-olds to measure health related quality of life in adolescents and young adults on the influence of age, gender, and method of administration. The measure has 36 close-ended questions of different types: Likert scales, rating scales and YES/NO options. These questions measure eight health concepts or domains: physical functioning [PF], role limitations due to physical health [RP], role limitations due to emotional problems [RE], energy/fatigue [EF], emotional well-being [EW], social functioning [SF], bodily pain [BP], and general health [GH]. There is also one single item dimension of health transition. Scoring is a 2-step process. At Step 1, pre coded numeric values are recorded on a scale of 0 [worst possible health state] to 100 [best possible health state] for each item. Therefore, a high score defines a more favourable health state. At Step 2 items in the same domain [health concept] are averaged.
