# Supplementary material for: DigiBete, a Novel Chatbot to Support Transition to Adult Care of Young People/Young Adults With Type 1 Diabetes Mellitus: Outcomes From a Prospective, Multimethod, Nonrandomized Feasibility and Acceptability Study
Source: JMIR Diabetes. 2025 Jul 23;10:e74032. doi: 10.2196/74032 (PMC12309419; doi:10.2196/74032)
Supplement: Multimedia Appendix 3 [file diabetes-v10-e74032-s003.docx]

**Multimedia Appendix- Supplementary File 3: Interview participants’ characteristics**

*Young people with T1DM interviewed from two sites.*

| **Age** | **Gender** | **Age at diagnosis** | **Years since diagnosis (calculated when recruited for feasibility)** | **Joint interview with parent** | **Gender of parent** |
| --- | --- | --- | --- | --- | --- |
| 15 | F | 7 | 8 | No | - |
| 15 | F | 5 | 8 | No | - |
| 15 | F | 7 | 8 | Yes | F |
| 13 | M | 2 | 11 | Yes | F |
| 13 | M | 4 | 9 | Yes | F |
| 11 | F | 10 | 1 | Yes | F |
| **Total number of YP = 6** | | | | | |

*Young adults with T1DM interviewed from two sites.*

| **Age** | **Gender** | **Age at diagnosis** | **Years since diagnosis (calculated when recruited for feasibility)** |
| --- | --- | --- | --- |
| 24 | F | 6 | 18 |
| 23 | F | 7 | 16 |
| 22 | F | 3 | 19 |
| 22 | F | 7 | 15 |
| 17 | F | 11 | 6 |
| 16 | F | 12 | 4 |
| **Total number of YA = 6** | | | |

*Four focus groups with T1DM Health Care Professionals from four sites.*

| **Profession** | **Number** |
| --- | --- |
| Play specialist | 1 |
| Specialist diabetes dietician | 1 |
| Dietician | 1 |
| Diabetes nurse | 6 |
| Diabetes nurse specialist | 1 |
| Paediatric diabetes nurse | 1 |
| Paediatric diabetes specialist nurse | 1 |
| Research nurse | 2 |
| Youth worker | 2 |
| Senor clinical psychologist | 1 |
| Clinical psychologist | 1 |
| Psychologist | 2 |
| Clinical support worker | 1 |
| Doctor | 1 |
| Diabetes Doctor | 1 |
| Consultant paediatrician | 2 |
| **Total number of HCPs = 25** | |
