# Supplementary material for: DigiBete, a Novel Chatbot to Support Transition to Adult Care of Young People/Young Adults With Type 1 Diabetes Mellitus: Outcomes From a Prospective, Multimethod, Nonrandomized Feasibility and Acceptability Study
Source: JMIR Diabetes. 2025 Jul 23;10:e74032. doi: 10.2196/74032 (PMC12309419; doi:10.2196/74032)
Supplement: Multimedia Appendix 4 [file diabetes-v10-e74032-s004.docx]

**Multimedia Appendix Supplementary File 4: Framework derived from analysis of qualitative data**

**Living with Type 1 diabetes**

- Managing it
- Psychosocial aspects

**Using the chatbot**

- How it is used
- Positive aspects of its functionality
- Perceived benefits of using the Chatbot

**Developing the chatbot**

- Appealing appearance
- Increasing functionality
- Range of content
