# Supplementary material for: DigiBete, a Novel Chatbot to Support Transition to Adult Care of Young People/Young Adults With Type 1 Diabetes Mellitus: Outcomes From a Prospective, Multimethod, Nonrandomized Feasibility and Acceptability Study
Source: JMIR Diabetes. 2025 Jul 23;10:e74032. doi: 10.2196/74032 (PMC12309419; doi:10.2196/74032)
Supplement: Multimedia Appendix 5 [file diabetes-v10-e74032-s005.docx]

*Supplementary File 5:* *Suggestions (wish list) for chatbot enhancement, extracted from participants’ qualitative responses*

| **Improve functionality by including:** | **Developers’ assessment of feasibility of implementing suggestions** | **Participants who requested this** |
| --- | --- | --- |
| Voice recognition for questions and responses | Yes | YP |
| Consideration of differing abilities of reading, writing and comprehension including dyslexia, visual, auditory learners | Yes | YP, HCP |
| Reduce misinterpretation of words used by people with diabetes like ‘low’ and ‘high’ | Yes | YP, Parent, HCP |
| Better accuracy on working out carbs and insulin dosage | To be further explored | YP |
| A widget to facilitate quicker access to Chatbot | Yes | YP, YA |
| Provide short text-based answers first before providing links to further information | Yes | YP, YA, Parent, HCP |
| Give users the choice to select short answer, longer answer and then links to further information | To be further explored | YA, HCP |
| Option to talk to a real person | Yes, if signposting to specific clinic | YA, HCP |
| Ability to link other long-term conditions | Yes, in a future development | YA |
| Provision of different languages | Yes, through language videos | HCP |
| Link to meals and carbs at food chain restaurants | Yes, but only through validated links | HCP |
| Direct users to their specialist team if their question is not answered provide link to their website with contact information | Yes | HCP |
| Ability to ‘rate’ or evaluate Chatbot responses for usefulness | Yes | HCP |
| Chatbot to pick up on Mental Health issues which are not overtly referred to e.g. ‘how do I get rid of my diabetes?’ may indicate the person is struggling with acceptance, this needs acknowledgement and referral to help. | Yes | HCP |
| Ability to differentiate between physical and mental health referral in an emergency | Yes | HCP |
| Ability to enter age (by user or HCP) to tailor responses to be more accurate and accessible | Yes | HCP |
| **Improving appearance** |  | **Participants who requested this** |
| Ability to design own avatar | Yes | YP, YA |
| **Building content** |  | **Participants who requested this** |
| Include alcohol advice | Yes | YP |
| Provide reassurance, psychological support | Yes | YA, HCP |
| Information needs to keep up to date | Yes | YA, HCP |
| Information on ‘libre’ | Yes | YA |
| Information on the effect of stress on diabetes | Yes | YA |
| Link to school care plan | Yes | Parent |
| Content related to going to festivals, abroad, driving | Yes | Parent |
| Eliminate incorrect answers and ensure appropriate links | Yes | HCP |
| Include advice around recreational drugs | Yes | HCP |

**Additional recommendations for future development include:**

- To prevent users from repeatedly inputting their identity or background story, it would be useful if the chatbot could ‘remember’ the user.
- If the chatbot could record details pertained to user backgrounds it would further assist the persuasive conversational capacity of the intervention. For example, if a user lives in a rural area, then suggestions to attend face-to-face social groups may be inappropriate.
- If the chatbot can accommodate user language choices in their conversational dialogue, it may enhance rapport.
- The chatbot could be improved if it could screen and record psychological factors that may impact the user’s ability to implement and sustain behaviour change recommendations.
